# A knowledge based approach to matching human neurodegenerative disease and animal models

**DOI:** 10.3389/fninf.2013.00007

**Published:** 2013-05-14

**Authors:** Sarah M. Maynard, Christopher J. Mungall, Suzanna E. Lewis, Fahim T. Imam, Maryann E. Martone

**Affiliations:** ^1^Department of Neurosciences, Center for Research in Biological Systems, University of CaliforniaSan Diego, San Diego, CA, USA; ^2^Genomics Division, Lawrence Berkeley National LaboratoryBerkeley, CA, USA

**Keywords:** phenotype, ontology, Neuroscience Information Framework, neurodegenerative disease, semantics

## Abstract

Neurodegenerative diseases present a wide and complex range of biological and clinical features. Animal models are key to translational research, yet typically only exhibit a subset of disease features rather than being precise replicas of the disease. Consequently, connecting animal to human conditions using direct data-mining strategies has proven challenging, particularly for diseases of the nervous system, with its complicated anatomy and physiology. To address this challenge we have explored the use of ontologies to create formal descriptions of structural phenotypes across scales that are machine processable and amenable to logical inference. As proof of concept, we built a Neurodegenerative Disease Phenotype Ontology (NDPO) and an associated Phenotype Knowledge Base (PKB) using an entity-quality model that incorporates descriptions for both *human* disease phenotypes and those of *animal models*. Entities are drawn from community ontologies made available through the Neuroscience Information Framework (NIF) and qualities are drawn from the Phenotype and Trait Ontology (PATO). We generated ~1200 structured phenotype statements describing structural alterations at the subcellular, cellular and gross anatomical levels observed in 11 human neurodegenerative conditions and associated animal models. PhenoSim, an open source tool for comparing phenotypes, was used to issue a series of competency questions to compare individual phenotypes among organisms and to determine which animal models recapitulate phenotypic aspects of the human disease in aggregate. Overall, the system was able to use relationships within the ontology to bridge phenotypes across scales, returning non-trivial matches based on common subsumers that were meaningful to a neuroscientist with an advanced knowledge of neuroanatomy. The system can be used both to compare individual phenotypes and also phenotypes in aggregate. This proof of concept suggests that expressing complex phenotypes using formal ontologies provides considerable benefit for comparing phenotypes across scales and species.

## Introduction

Model systems are the cornerstones of translational research. The number of model organisms is increasing, as governments and other agencies have invested heavily in the creation of comprehensive panels of genetically modified organisms for the study of disease (Guan et al., 2010; Ringwald and Eppig, 2011). Building informatics resources that make maximal use of these model systems by promoting discovery of appropriate systems and comparisons among them has been challenging. The need for such systems has been recognized by the model organism databases (Blake et al., 2011; Shimoyama et al., 2011) and programs such as the Link Animal Models to Human Disease Initiative [LAMHDI (http://lamhdi.org)]. Such systems seek to provide the means to select appropriate animal models for analysis and to identify common mechanisms across conditions. These systems generally make use of homologous genes to relate models (e.g., LAMHDI), or contain assertions that animal X is a model of disease Y (e.g., Mouse Genome Database; Blake et al., 2011).

The above approaches, while valuable, are limited by the complexity of disease-related phenotypes, particularly in the nervous system with its myriad of cell types and functional systems. The relationship between gene and model system and disease phenotype is not straightforward (Strohman, 2002; Houle et al., 2010; Doelken et al., 2013), even when the gene(s) underlying a disease are known. At the most basic level, molecular networks are involved in multiple processes within a cell and because the spatial and temporal organization of molecular networks may differ across cell types, body systems and organisms, mutations in a given gene may give rise to multiple phenotypes. This phenomenon is commonplace in neurodegenerative diseases where the affected gene may be widely expressed, while the regional and cellular pathology are much more restricted. Conversely, mutations in multiple genes contained within similar pathways or cell types may give rise to the same phenotype at a macromolecular scale.

Selection of appropriate models and mining of model systems to look for common molecular pathways related to human disease requires more effective means for searching for and comparing phenotypes. However, phenotypic descriptions tend to represent combinations of complex structural features and physiological processes spanning multiple spatial and temporal scales, often involving multiple anatomical or functional systems. Comparison among phenotypes, even for relatively straightforward examples, usually requires a significant amount of human knowledge and effort (Gupta et al., 2003). Consider a statement in one study that *pigmented cells in the substantia nigra degenerate in the human* and in another study that *tyrosine hydroxylase-expressing cells in the mouse are decreased in number*. Understanding that these two phenotypes are highly similar requires the knowledge that: (1) pigmented cells in the substantia nigra of human express dopamine; (2) that tyrosine hydroxylase is a marker for dopaminergic cells; and (3) that “degenerated” and “decreased in number” are related to one another, in that degeneration at the tissue level often involves decrease in number of cells. Such comparisons are also confounded by the cultural practices of different populations of researchers leading to the use of different vocabularies for describing their results. These differences may be particularly significant when crossing between clinical terminologies used to describe patients and basic science vocabularies used to describe phenotypes in experimental assays.

Because of their inherent complexity, most descriptions of phenotypes in the literature or on-line databases are currently expressed in free text. Free text is notoriously difficult to parse, even for well-defined entities such as genes and organisms (Washington et al., 2009), making free-text phenotype descriptions largely opaque to modern computational methods. In order to provide a more effective means for comparing phenotypes among organisms, groups have been working on the development of formal ontologies for phenotype descriptions. An ontology encodes human knowledge by defining concepts and relationships within a domain in a way that makes them machine processable (Munn and Smith, 2008). Ontologies such as the Mammalian Phenotype Ontology (MPO; Smith et al., 2005b) and the Human Phenotype Ontology (Robinson and Mundlos, 2010) provide formal descriptions of phenotypes in human and model systems. Phenotype descriptions are arranged in hierarchies so that information systems can use the ontology to group related phenotypes under common subsumers, e.g., “degenerated substantia nigra” is a type of “abnormality of the midbrain.”

The existing community phenotype ontologies are designed to provide broad coverage of major phenotypes associated with mammalian model systems and human diseases. However, the available phenotype annotations are often not of sufficient granularity to cover the array of affected entities and relative quantitative phenotypes encountered during an experimental investigation, particularly for the varied and heterogenous phenotypes encountered in the nervous system. For example, the Mouse Genome Informatics (MGI) database (Blake et al., 2011) annotation of Tg(Prnp-SNCA^*^A53T)83Vle (a transgenic mouse incorporating a human alpha synuclein variant implicated in Parkinson's Disease) described in Giasson et al. (2002) includes the MPO term “alpha-synuclein inclusion body” (MPO identifier MP:0008493^1^) but uses free text to provide additional detail, such as the fact that the inclusion bodies are observed in the spinal cord, brainstem, cerebellum, and thalamus. An information system thus looking for mice that have abnormal cellular aggregates in cerebellar cells would have to parse a considerable amount of text and infer knowledge that inclusion bodies are contained within cells. Ideally the phenotype annotation would provide a computable means of linking to the anatomical systems affected, and the qualitative nature of the change in these systems.

The need for tools for flexible phenotype descriptions led to the construction of the Phenotype and Trait ontology (PATO; Gkoutos et al., 2005). PATO is an ontology of qualities that can be combined with any independent ontological entity to provide a formal description of a phenotype (Mungall et al., 2010; Chen et al., 2012). For example, statements such as *swollen mitochondrion* can be represented as an entity that is a kind of “mitochondrion” (Gene Ontology identifier GO:0005739) that has a “swollen” (PATO:0001851) quality. This EQ (entity + quality) approach can be extended to include the types of nested and relational expressions typically encountered in an experiment, e.g., that *neurons in the substantia nigra have increased numbers of abnormal protein aggregates compared to neurons in the locus coeruleus*. If these EQ descriptions are expressed using a logical formalism such as the Web Ontology Language (OWL) then we can use automated reasoners to compute relationships between them (Mungall et al., 2010). When combined with semantic-similarity based algorithms, we can measure the degree of similarity between two entities based on their phenotype. An example of a system that combines reasoning and semantic similarity over large phenotypic datasets is the Mouse Finder, which has previously been used to link animal models to human diseases (Washington et al., 2009; Chen et al., 2012).

In this study, we explored the use of the EQ approach to the challenging problem of representation and comparison of structural phenotypes encountered in neurodegenerative disease and associated animal models. This work was motivated by the need to develop annotation tools for microscopic imaging data available through the Cell Centered Database (http://ccdb.ucsd.edu) and other imaging databases, to ensure that the content of these images are exposed in a way that facilitates interlinking with other data through a common semantic framework (Lam et al., 2007; Imam et al., 2012). We built an ontology of multi-scale structural phenotypes observed in humans with a disease and animal models. We have used PATO in conjunction with the Neuroscience Information Framework (NIF) ontologies (Bug et al., 2008; Imam et al., 2012) for the neurological entities. The NIF Standard (NIFSTD) is a set of modular ontologies built largely from community ontologies covering the major domains of neuroscience, including diseases, brain regions, cells, and subcellular parts. Relations between the entities of these modules (e.g., cell type to brain region, or cell type to neurotransmitters or markers such as tyrosine hydroxylase) are contained within separate bridging modules, providing an integrated multiscale view of entities of relevance to neuroscience (Imam et al., 2012). We then employed a combination of reasoning and semantic similarity methods, using the PhenoSim framework (previously implemented as part of OBD; Washington et al., 2009) to compute similarities among phenotypes. We show that the system is capable of representing diverse phenotypes using a flexible grammar and using knowledge encoded within community ontologies to retrieve relevant phenotypes across anatomical scales and organisms. Finally, we show that this approach lays the groundwork for designing a system to effectively match animal models to human disease based on structural phenotypes.

## Materials and methods

### Ontology construction

NDPO and PKB were constructed using Protégé 3.3.1 and 4.0 (http://protege.stanford.edu/). Both NDPO and PKB use the Basic Formal Ontology as an upper ontology and relations from the OBO Relations Ontology (Smith et al., 2005a), following the pattern laid down in (Mungall et al., 2010).

NDPO imports the NIFSTD ontology for anatomical entities and PATO. The NIFSTD in turn imports many community ontologies or contains cross-references to them (Mungall et al., 2010). When necessary, additional entities were added to the base modules, and additional relations were added between modules in the form of bridge files (Imam et al., 2012). We used the OWL version of PATO generated by the OBO Foundry (http://purl.org/obo/owl/PATO). Organisms and phenotypes are modeled in the NDPO as OWL class level expressions. For example, the class “Human with Alzheimer's disease” is specified in OWL as:

Class: “Human with Alzheimer's disease”

EquivalentTo: Human and bearer_of some “Alzheimer's disease”

…

Each of the individual phenotypes is then defined using equivalence axioms (see Table 1 for examples). See Horridge (2011) for an explanation of terminology related to building and using ontologies.

**Table 1:** **Phenotype representations from NDPO involving the substantia nigra of a representative individual with parkinson's disease**.

**Textual description**	**OWL expression and EQ expression**	**References**
Neurons decreased in number in the substantia nigra pars compacta	“Has fewer parts of type” and “inheres in” some “substantia nigra pars compacta” toward some neuron	PMID:12971891
Neurons decreased in number in the substantia nigra	“Has fewer parts of type” and “inheres in” some “substantia nigra and toward some neuron”	PMID: 9617789
Dopaminergic cells decreased in number in the substantia nigra	“Has fewer parts of type” and toward some “substantia nigra dopaminergic cell”	Not recorded
Substantia nigra dopamine cells decreased in number	“Has fewer parts of type” and toward some “substantia nigra dopaminergic cell”	Not recorded
Degeneration of substantia nigra dopaminergic cells	Degenerate and “inheres in” some “substantia nigra dopaminergic cell”	PMID: 11253364
Dopaminergic cells containing neuromelanin decreased in number in the substantia nigra pars compacta	“Has fewer parts of type” and toward some “substantia nigra dopaminergic cell” and “has part” neuromelanin	PMID: 19086884
Substantia nigra pars compacta depigmentation	“Has fewer parts of type” and “inheres in” “substantia nigra pars compacta” toward some neuromelanin	PMID: 12971891
Substantia nigra pars compacta decreased in volume	“Decreased volume” and “inheres in” some “substantia nigra pars compacta”	PMID: 17978822
Substantia nigra pars compacta degenerated	Degenerate and “inheres in” some “substantia nigra pars compacta”	PMID: 16772866
Substantia nigra decreased in volume	Decreased volume and “inheres in” some “substantia nigra”	PMID: 18941719
Atrophy of midbrain	Atrophied and “inheres in” some midbrain	PMID: 18941719
Midbrain degenerated	Degenerate and “inheres in” some midbrain	PMID: 20308987

The first column shows a succinct textual summary of the phenotype, typically extracted directly from the paper (reference). The second column shows the OWL class expression (written in Manchester Syntax) used to define each phenotype. The design pattern from Mungall et al. (2010) is used, with PATO used for the phenotypic quality, and NIFSTD used as a source of entities. The relation “inheres in” is used to represent the affected entity, and for relational qualities the “toward” relation is used to indicate the additional entity. Thus, “‘has fewer parts of type’ and inheres in some substantia nigra pars compacta and toward some neuron” translates to “Neurons are decreased in number in the substantia nigra pars compacta (column 1).”

Each class in NDPO is identified via a globally unique and meaningless identifier expressed as a URI, and is also assigned a human readable label. For ease of reading, we use these human readable labels in this report.

### Rationale for design

The NDPO is not designed to function as a diagnostic system, but as a means to look for common phenotypes occurring among organisms that are linked to processes relevant to disease. Thus, no particular weighting is given to hallmark features of a disease, nor did we attempt to create deep models of the disease process. We followed the basic model established by the Ontology of General Medical Science (Scheuermann et al., 2009) by treating disease as a dependent continuant. A dependent continuant cannot exist independently of the entity that bears it. We thus modeled structural phenotypes observed in humans that bear a disease, rather than modeling the disease itself (Gupta et al., 2003; Scheuermann et al., 2009).

### Phenotype similarity calculation

We used a variation of the methods described in Washington et al. (2009) for finding organisms that were closely related via similar phenotypes. We ported the original implementation (which relied on a relational database backend) to a new system that uses the OWL API (Horridge and Bechhofer, 2009) called OWLSim (available from: http://code.google.com/p/owltools/wiki/OwlSim). We configured this to work with our phenotype descriptions, and call this configuration “PhenoSim.”

The OWLSim comparison metrics rely on the inferred attributes shared between any two given phenotypes. The inferred attributes of a phenotype are the set of classes in the reflexive transitive closure of the phenotype, calculated by recursively following all OWL equivalence or subclass axioms to either classes or anonymous class expressions. We extend the path from class intersections to each element of the intersection, and each existential restriction to the filler expression. This can be expressed more formally as the construction of a graph of pairwise edges <X,Y> from the total set of axioms and class expressions in an ontology:

1. Add edge <X,Y> for every SubClassOf(X,Y) or EquivalentClasses(X,Y) axiom
2. Add edge <X,Y> if there exists a class expression X, and X = ObjectIntersectionOf(L), and X is a member of L
3. Add edge <X,Y> if there exists a class expression X, and X = ObjectSomeValuesFrom(part_of,Y)

We calculate the set of inferred attributes from a phenotype P by finding the closure of all edges emanating from P, including P in the set.

Prior to calculating the closure of each phenotype, we pre-reasoned the combined ontology and asserted all directly inferred axioms.

We use two methods for computing the similarity between any two phenotypes—the Jaccard Similarity (SimJ), and the information content (IC) of the Least Common Subsumers (LCS). The SimJ between two phenotypes *p* and *q* is the ratio of shared attributes vs. total attributes, and is calculated as:

$${\text{sim}}_{\text{J}}(p,\;q)=\frac{\left|a^{p}\cap a^{q}\right|}{\left|a^{p}\cup a^{q}\right|}$$

where *a*^*p*^ is the inferred attributes of phenotype *p*.

The second method for measuring the similarity of two phenotypes is IC based, and provides a measure of how unusual or “surprising” the set of attributes in common is. We first define the LCS phenotype of a pair of phenotypes *x* and *y* as the most specific set of all shared attributes:

$$\text{LCS}(x,\;y)=\{a|a\in a^{x}\cap a^{y},\;\neg \exists a^{\prime }:\left[a^{\prime }\in a^{x}\cap a^{y},\text{ path}(a,\;a^{\prime }),\;\neg \text{path}(a^{\prime },\;a)\right]\}$$

here, path(*a*,*a'*) holds if there is a reflexive path in the graph between *a* and *a'*, as described above. The LCS can be thought of as the most compositional description of the phenotype that subsumes x and y. To calculate the IC of the LCS we need to know the set *OP*, which is the set of all pairwise mappings between organisms and phenotypes

$$\text{ICS}(x,\;y)=-\log_{2}\left(\frac{\left|\{o|(o,\;p)\in OP,\text{ sub}(p,\text{ LCS}(x,\;y))\right|}{\left|\{o|(o,\;p)\in OP\}\right|}\right)$$

The relation sub(*p*, *A*) holds if *p* is subsumed by the conjunction of attribute set *A*:

$$\text{sub}(p,\;A)\Leftrightarrow \neg \exists a\in A,\;a\notin p^{a}$$

The IC is higher for less frequent LCS's. Thus, a match in which the combination of attributes held in common is rare, or involves highly granular terms, will score more highly than those involving more frequent terms or less granular terms.

Both IC and SimJ give a measure of similarity between a pair of *phenotypes*. We are interested in similarity at the organism level, and an organism can have multiple phenotypes, so we needed a means of aggregating the set of all phenotype pairs for each of the two organisms. We use two methods—taking the maximum score from the set of all phenotype pairs, and finding the average of the best matching pairs. We use the prefix Max and Avg for each of these respectively.

We focus on 4 organism-matching scores in particular:

The MaxIC is the maximum IC of the best matching pair of phenotypes:

$$\text{MaxIC}(o^{1},\;o^{2})=\max\{s|(o^{1},\;p^{1})\in OP,\;(o^{2},\;p^{2})\in OP,\;s=\text{ICS}(p^{1},\;p^{2})\}$$

The MaxSimJ is the maximum SimJ for the best matching pair of phenotypes:

$$\text{MaxSimJ}(o^{1},\;o^{2})=\max\{s|(o^{1},\;p^{1})\in OP,\;(o^{2},\;p^{2})\in OP,\;s=\text{SimJ}(p^{1},\;p^{2})\}$$

The AvgIC is the average IC of all best-matching pairs of phenotypes:

$$\begin{array}{l} \text{ AvgIC}(o^{1},\;o^{2})=\text{avg}\{s|\left(o^{1}p^{1}\right)\in OP,\;(o^{2},\;p^{2})\in OP,\\ \;s=\text{ICS}(p^{1},\;p^{2}),\\ \left((\neg \exists p^{3}:o^{1},\;p^{3})\in OP,\text{ ICS}(o^{1},\;p^{3})>s)\wedge (\neg \exists p^{3}:o^{2},\;p^{3}\right)\\ \;\in OP,\text{ ICS}(o^{2},\;p^{3})>s))\} \end{array}$$

The AvgSimJ is the average SimJ of all best-matching pairs of phenotypes:

$$\begin{array}{l} \text{AvgSimJ}(o^{1},\;o^{2})=\text{avg}\{s|(o^{1},\;p^{1})\in OP,\;(o^{2},\;p^{2})\in OP,\\ \;s=\text{SimJ}(p^{1},\;p^{2}),\\ \;((\neg \exists p^{3}:o^{1},\;p^{3})\in OP,\text{ ICS}(o^{1},\;p^{3})>s)\wedge (\neg \exists p^{3}:o^{2},\;p^{3})\\ \;\in OP,\text{ SimJ}(o^{2},\;p^{3})>s))\} \end{array}$$

From these we derive a combined score:

$$\text{Combined}(o^{1},\;o^{2})=\text{AvgSimJ}(o^{1},\;o^{2})+\text{AvgSimJ}(o^{2},\;o^{1})+\text{MaxIC}(o^{1},\;o^{2})$$

### Web-based front end

We created a web interface for browsing organisms, diseases, phenotypes, and phenotype matches called the PKB-Browser, available at (http://berkeleybop.org/pkb/). This interface provides query capability through key word searches, and allows the user to browse and compare the contents of the knowledge base without the need to know formal query languages like SPARQL.

The PKB Browser is constructed using the Thea OWL Library (Vassiliadis et al., 2009) and Clio Patria (http://cliopatria.swi-prolog.org/home). The PKB browser is a prototype interface that is freely available but is in beta release. We are currently in the process of ingesting the data using NIF DISCO data integration framework [Marenco et al., 2010] and porting the user interface to the Monarch Initiative (http://monarchinitiative.org/) platform.

## Results

**Figure 1:**
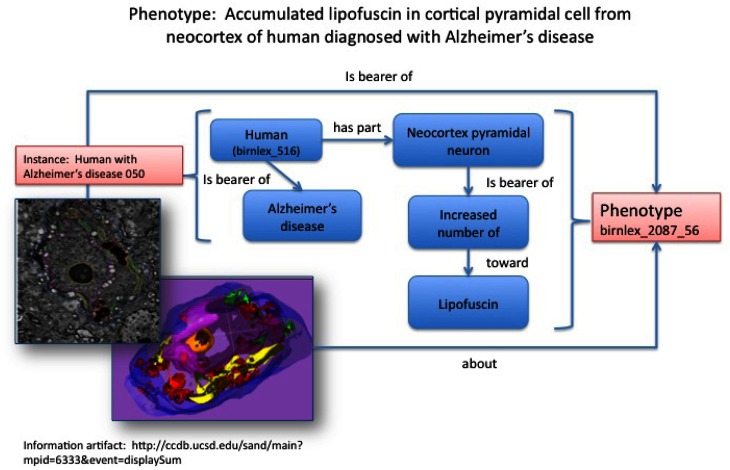
**Structured phenotype description derived from imaging data stored in the CCDB (Accession #: MP6333).** The phenotype statement describing lipofuscin accumulation in a pyramidal cell from the cerebral cortex of a patient with Alzheimer's disease is shown at the top. The corresponding image is shown on the left. The entire phenotype is recorded as a series of statements, each depicted as a relationship (arrow) between two classes (boxes). Each of the classes are derived from the NIFSTD ontologies; for ease of understanding, the preferred label is displayed rather than the numerical class name (e.g. birnlex_516 for “Human”). Classes are displayed in blue while instances are displayed in pink.

To enable comparison of phenotypes across species and scale, we constructed: (1) a Neurodegenerative Disease Phenotype Ontology (NDPO) focused on human disease; and (2) the Phenotype Knowledge Base (PKB), describing phenotypes observed primarily in model organisms. Both of these resources use PATO and the NIFSTD ontologies as building blocks to create more complex expressions. The original NDPO was developed to create tools for structured annotations of microscopic imaging data contained in the Cell Centered Database [(http://ccdb.ucsd.edu) Figure 1] and Whole Brain Catalog (http://wholebraincatalog.org/). As the number of publically available data on neurodegenerative disease within these resources is still small, we used phenotypes reported in the literature to develop and test the model, focusing on phenotypes involving structural alterations at the tissue, cellular, and subcellular levels and excluding descriptions of dynamic processes. Both NDPO and PKB are encoded in OWL; NDPO consists primarily of class-level logical statements about the types of entities affected, whereas PKB consists primarily of instance-level statements about specific organisms that exhibit the phenotypes. We thus refer to NDPO as an ontology and to PKB as a knowledge base that uses NDPO, noting that from a formal OWL perspective both are ontologies.

The model of NDPO/PKB is organism-centric; that is, all phenotypes are borne by a particular organism (Figure 1). The model does not include any explicit assertions about the relationship of a phenotype to a disease process; rather diseases and phenotypes are correlated through the agency of the organism.

### The neurodegenerative disease phenotype ontology (NDPO)

The NDPO (http://ontology.neuinfo.org/NDPO/NDPO.owl) contains statements associating neurodegenerative diseases with their characteristic phenotypes. These statements were curated from multiple literature sources, and entered into NDPO using a standard template. We assigned phenotypes to reference entities representing a “composite” human with a particular disease, e.g., *Human with Parkinson*'*s disease*, bearing phenotypes typically associated with that disease. These phenotypes represent generally accepted features of a particular disease, such as would be contained within a review article.

### Phenotype knowledge base (PKB)

The PKB (http://ontology.neuinfo.org/NDPO/PKB.owl) contains statements associating individual organism instances with phenotypes observed in those organisms. We collected statements for both humans and animal models of neurodegenerative diseases, using both scientific reports and CCDB images as sources. Instance representations model a single individual in which a set of phenotypes was observed, such as was described in a single scientific report or a particular image set. The same organism may have multiple phenotypes associated with it, provided that they were contained within a single report, and there may be multiple instances of the same organism. Organisms are represented as instances of a class from the NIFSTD Organism ontology module, which includes a Linnean taxonomy that goes down to the level of individual strains. Each organism instance is provided with a label such as *Human with Alzheimer's disease 050* or *Sprague-Dawley rat 528*. Where possible, we utilize the official strain nomenclature provided by the Mouse Genome Institute for mice (Linder, 2003). However, as these names are intricate and lengthy, each mouse is also given a simple descriptive preferred label for ease of reference.

Because neurodegenerative diseases evolve over time, we encountered many phenotype descriptions that were linked to a particular stage of a disease process or age of an organism. To handle this case, we created temporal slices of an organism at each time or particular stage, to represent each distinct temporal phenotype, e.g., PS19 mouse at age 60 days. However, in this version of the PKB, no relationships are drawn among these temporal slices; they are treated as separate organism instances.

Phenotypes were entered by various individuals, including the authors of this paper, and summer student interns who worked under our supervision. As is common with ontology development and annotation, we evolved both the ontology representation and our annotation standards over time. When possible, we tried to back propagate these changes to the NDPO/PKB, but, as is also common with information systems, we still find inconsistencies. As the NDPO/PKB were built from the NIFSTD ontologies, which itself imports community ontologies, the base ontologies are undergoing constant evolution. For the analyses reported here, we used the versions of the ontologies stated in the methods.

### Combined knowledge base and browser

The combination of PKB and NDPO contains 1260 phenotype statements for 11 representative humans with disease and 90 models, representing macromolecular, subcellular, cellular, and gross anatomical characterization of features observed in structural studies of these organisms (available at: http://berkeleybop.org/pkb/organisms). Papers were chosen to include a representation of structural phenotypes across scales for brain and spinal cord. Of the 11 human diseases modeled, we included explicitly asserted models for 6 of them. The majority of non-human phenotypes were derived from mouse models, comprising 34 individuals, representing 14 distinct mutants. Our goal in selecting papers for inclusion was not to include a random sample, but to ensure that the phenotype model could accommodate the range of phenotypes reported by basic and clinical researchers, and to test our ability to retrieve related phenotypes based on region, cell types, subcellular structure, or macromolecular constituent. Thus, we ensured that the knowledge base included related phenotypes across scales. As many neurodegenerative disorders are characterized as synucleopathies, we included multiple animal models with modifications to one or more forms of synuclein, as these often display unique phenotypes. We constructed a web-based browser called the Phenotype Knowledge Browser; (http://berkeleybop.org/pkb/) that provides a collection of views on top of the underlying OWL ontologies, allowing users to navigate between diseases, organisms, and phenotypes.

**Figure 2:**
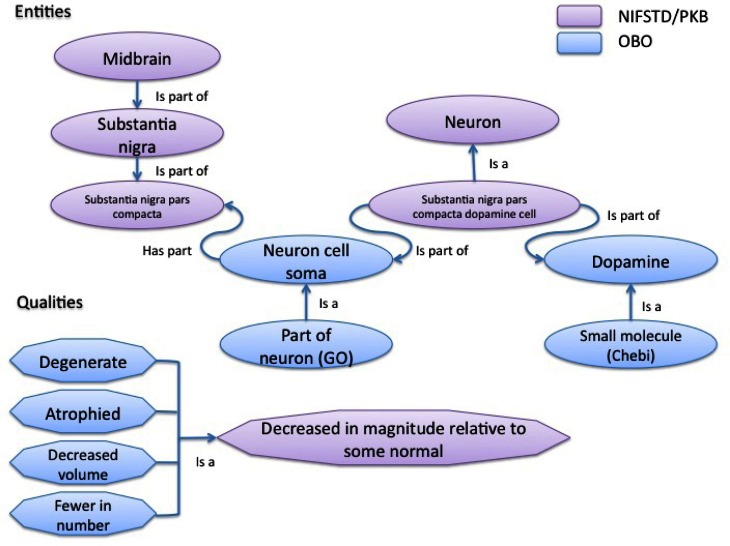
**Schematic of entity and quality relationships coded in the core ontologies used to build the NDPO to provide the requisite knowledge for comparing the phenotypes in Table 1.** NIFSTD is built in a modular form with each module covering a single domain of neuroscience, e.g., gross anatomy, subcellular entities, cells, and molecules. NIFSTD has been building bridge ontologies that span one or more of these modules. For example, the NIFSTD cell bridge relates neurons to brain regions and molecular entities. Entities drawn from the NIFSTD are shown in purple. NIFSTD itself imports or cross references several community ontologies from the Open Biological Ontologies (OBO) Foundry. These ontologies include Chemicals of Biological Interest (Chebi), Gene Ontology (GO), and the PATO qualities.

Examples of typical phenotype annotations are shown in Table 1, which extracts phenotype observations regarding the substantia nigra from NDPO. As is evident, even with a more structured representation, similar phenotypes are expressed in multiple ways and at different anatomical scales, e.g., *substantia nigra pars compacta degenerates* vs. *substantia nigra degenerates*, reflecting the diversity of descriptions in the literature. Different formulations of the same entities and qualifiers, e.g., *dopaminergic cells in the substantia nigra are decreased in number* vs. *substantia nigra dopaminergic cells are decreased in number* were coded as identical OWL statements when appropriate and where the meaning of the author could be ascertained. When the exact meaning could not be ascertained from similar entities or qualities, e.g., degenerate, decreased in number, decreased in volume, the original qualifiers were generally retained, leading to multiple expressions of similar phenotypes. Additional classes and relationships were added to the NIFSTD ontologies when required in order to provide the basic knowledge required to express or compare phenotypes. Figure 2 illustrates some of the types of entities and relationships between them that are required for comparing many of the phenotypes listed in Table 1.

### Comparing phenotypes

We used PhenoSim to compute and rank pairwise comparisons between organisms, based on the semantic similarity and IC between their associated phenotypes, as detailed in the methods. The scores can be partitioned into 3 sets: reference disease vs. reference disease, reference vs. model, and model vs. model. Thus, PhenoSim can be used to compare phenotypes from human disease to human disease; human disease to animal model and animal model to animal model. These pairings were then loaded into the PKB browser.

We tested the ability to provide meaningful matches of both individual and aggregate phenotypes by issuing a series of competency questions via the PKB browser as described below. The competency questions were designed to test whether PKB was able to use the relationships recorded in the ontology to improve retrieval compared to string search alone. Because PhenoSim computes similarity and IC based on common subsumers, every phenotype could potentially be comparable to another at a gross level, just by virtue of them referencing some structure in the brain. Thus, we evaluated the results as to whether the reasoning process produced results that would be meaningful to someone knowledgeable in the domain (author Maryann E. Martone), the number of false negatives and positives, and whether the scores used to rank the similarity (Similarity score) and meaningful (IC) were reliable predictors of the relevancy. In the following, we go through specific examples of four types of use cases:

Use case 1: Find an individual phenotype (Q1)
Use case 2: Find organisms that share a particular phenotype (Q2)
Use case 3: How do phenotypes compare overall for any two organisms (Q3)
Use case 4: Which organisms share the most similarity across all phenotypes to a given disease? (Q4)

Query 1 (Q1): Which organisms have phenotypes involving GABAergic neurons/cellular inclusions?

**Figure 3:**
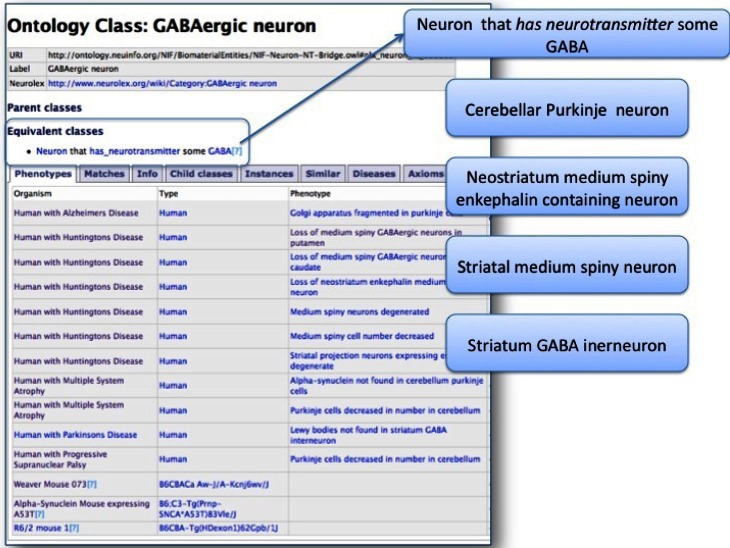
**Results of query for all phenotypes involving GABAergic neurons (Q1).** Because the NIFSTD ontology provides classifications of neurons by their neurotransmitters, PhenoSim is able to retrieve phenotypes where GABA is not explicitly mentioned in the statements. All of the neurons listed on the right use GABA as a neurotransmitter and are thus correctly returned as GABAergic neurons.

PKB allows users to query for phenotypes involving a particular entity, returning a list of organisms with phenotypes involving that entity (Figure 3). For Q1, as expected, PKB returned phenotypes containing explicit reference to GABAergic neurons, e.g., *loss of medium striatal GABAergic neurons in the caudate nucleus of a human with Huntington*'*s disease*. As shown in Figure 3, however, the NIFSTD ontology defines GABAergic neuron as the equivalent class *Neuron that uses neurotransmitter GABA*. Thus, PKB also returns phenotypes associated with neuron classes for which the neurotransmitter is known to be GABA, e.g., cerebellar Purkinje neurons, neostriatal medium spiny neurons, regardless of whether GABA is explicitly mentioned. Note also that PKB contains negative statements such as *Lewy bodies are not found in striatal GABA interneurons*, because the type of cells that are spared in neurodegenerative disease is an important piece of information, both for capturing the total phenotype of the disease and for looking at possible mechanisms of selective vulnerability. At present, however, there is no way to refine the query to obtain only the positive associations.

As a comparison, we searched for “GABAergic neuron” in the Mammalian Phenotype and Human Phenotype ontology through the NCBO Bioportal search function. The MPO returned “abnormal GABAergic neuron morphology MP:0003245” with child “loss of GABAergic neuron; MP:0003246.” Although the MPO contains numerous phenotypes involving types of GABAergic neurons, e.g., Purkinje cells, medium spiny neuron, only one specific cell type using GABA as a neurotransmitter was returned, due to a reference to GABAergic neuron in the definition: “abnormal olfactory bulb periglomerular cell morphology; MP:0009943.” No explicit relationship was recorded between the more generic “GABAergic neuron” and the specific types of cells using GABA as a neurotransmitter. Similarly a query of PKB for “cellular inclusion,” retrieved phenotypes for Lewy bodies, cellular inclusions, neurofibrillary tangles, Pick bodies, among others, while MPO returned only for the string “cellular inclusion.”

Q2: Which organisms share phenotypes involving pyramidal cells?

This query depends on the semantic similarities between pairs of phenotypes computed by PhenoSim. The results include all phenotype matches that involve the class *Pyramidal cell*, or a subclass or part of a pyramidal cell. The phenotype matches are computed for all organisms, whether model or reference humans. Representative results for this query are shown in Table 2, using the SimJ measure between pairs of phenotypes, which scores each pairing between 0 (no similarity) and 1 (no discernable difference). For example, the first match shown in Table 2 is between a human with Amyotrophic Lateral Sclerosis (ALS) and a human with Multiple System Atrophy (MSA) both of which involve a reduction in the number of Betz cells, a large pyramidal cell found in motor cortex. In this case, the two phenotype descriptions are identical, so the score is 1.

**Table 2:** **Comparison of pyramidal cell phenotypes among all organisms**.

**Least common subsumer**	**SimJ**	**Organism A**	**Phenotype A**	**Organism B**	**Phenotype B**
Betz Cell ^*^ has fewer parts of type	1	Human with Amyotrophic Lateral Sclerosis	Betz Cell ^*^ has fewer parts of type	Human with Multiple System Atrophy	Betz Cell ^*^ has fewer parts of type
Neocortex pyramidal cell layer 5 ^*^ has fewer parts of type	0.944	Human with Amyotrophic Lateral Sclerosis	Betz Cell ^*^ has fewer parts of type	5XFAD transgenic mouse 2	Neocortex pyramidal cell layer 5 ^*^ has fewer parts of type
Neocortex pyramidal cell layer 5 ^*^ has fewer parts of type	0.944	Human with Multiple System Atrophy	Betz Cell ^*^ has fewer parts of type	5XFAD transgenic mouse 2	Neocortex pyramidal cell layer 5 ^*^ has fewer parts of type
Pyramidal cell ^*^ has fewer parts of type	0.882	Human with Huntingtons disease	Pyramidal cell ^*^ has fewer parts of type	5XFAD transgenic mouse 2	Neocortex pyramidal cell layer 5 ^*^ has fewer parts of type
Pyramidal cell ^*^ has fewer parts of type	0.833	Human with Amyotrophic Lateral Sclerosis	Betz Cell ^*^ has fewer parts of type	Human with Huntingtons disease	Pyramidal cell ^*^ has fewer parts of type
Pyramidal cell ^*^ has fewer parts of type	0.833	Human with Huntingtons disease	Pyramidal cell ^*^ has fewer parts of type	Human with Multiple System Atrophy	Betz Cell ^*^ has fewer parts of type
Neocortex pyramidal cell layer 5 Protein ^*^ has number of	0.714	5XFAD transgenic mouse 1	Neocortex pyramidal cell layer 5 ^*^ Beta-Amyloid ^*^ has number of	R6/2 mouse 3	Neocortex pyramidal cell layer 5 ^*^ Huntingtin ^*^ has extra parts of type
Neocortex pyramidal cell layer 5 normal ^*^ altered number of	0.682	5XFAD transgenic mouse 2	Neocortex pyramidal cell layer 5 ^*^ has fewer parts of type	R6/2 mouse 3	Neocortex pyramidal cell layer 5 ^*^ Huntingtin ^*^ has extra parts of type
Neocortex pyramidal cell layer 5 ^*^ normal ^*^ altered number of	0.652	Human with Amyotrophic Lateral Sclerosis	Betz Cell ^*^ has fewer parts of type	R6/2 mouse 3	Neocortex pyramidal cell layer 5 ^*^ Huntingtin ^*^ has extra parts of type
Neocortex pyramidal cell layer 5 ^*^ normal ^*^ altered number of	0.652	Human with Multiple System Atrophy	Betz Cell ^*^ has fewer parts of type	R6/2 mouse 3	Neocortex pyramidal cell layer 5 ^*^ Huntingtin ^*^ has extra parts of type
Pyramidal cell Regional part of hippocampal formation has fewer parts of type	0.645	Human with Brain ischemia	Hippocampus CA1 pyramidal cell ^*^ has fewer parts of type	5XFAD transgenic mouse 2	Pyramidal cell ^*^ Subiculum ^*^ has fewer parts of type

A selection of phenotype comparisons involving pyramidal cells, selected from a larger set returned by PKB. The Least Common Subsumer (LCS) column shows the set of attributes that are shared in common between Phenotype A from Organism A and Phenotype B from Organism B. A pairwise similarity score (SimJ) is calculated for each match, with 1 being an exact match. For readability, only the entities and qualities are shown separated by an

* *, rather than the OWL expression*.

Similarity is determined in part on the number of shared metrics between any two phenotypes, utilizing the asserted and inferred relationships for the entities/qualities in the underlying ontologies. Thus, the second highest scoring match in Table 2 is between a phenotype involving a reduction in Betz cells in a Human with ALS and a reduction of cells in *Neocortex pyramidal cell layer 5* in a 5XFAD transgenic mouse. The match is high scoring, as Betz cells are a type of layer 5 pyramidal cell, but scores less than would an exact match. In this case, the least common subsumer that relates these pairs is *Neocortex pyramidal cell layer 5 has fewer parts of type.* Note that the more generic phenotype *Pyramidal cell has fewer parts of type*, i.e., there is a reduction in the number of pyramidal cells, in a Human with Huntington's disease (result 4), is ranked slightly lower in similarity to a reduction in neocortex pyramidal cell layer 5 neurons in the 5XFAD transgenic mouse 2 because pyramidal cell is a more generic subsumer than a neocortex pyramidal cell.

Q3: How does the phenotype of a weaver mouse compare to a patient with Huntington's disease?

The PKB browser lets a user compare the phenotypes of any two organisms within the database by providing a list of phenotypes matched according to the least common subsumer. Matches are ranked according to overall similarity (SimJ) and to the IC, a reflection in part of the granularity of the common subsumer. A single phenotype can match to more than one phenotype, based on different subsumers.

The results for the comparison of the weaver mouse, a murine mutant with several motor defects and degeneration of the substantia nigra and cerebellum, and a human with Huntington's Disease, also characterized by motor disturbances and damage to the basal ganglia, is shown in Table 3. Although there is no presumed genetic linkage between the two organisms, they do share some functional similarities that reflect damage to common systems of the brain. For this use case, we examined the pairwise matches returned from PhenoSim and classified them according to whether they were (1) Plausible and useful (green); (2) Plausible but not useful (orange); (3) Neither plausible or useful (white). For those phenotypes for which PhenoSim returned no matches, we ranked them as to whether we would have matched them with another phenotype based on our knowledge of anatomy.

**Table 3:**
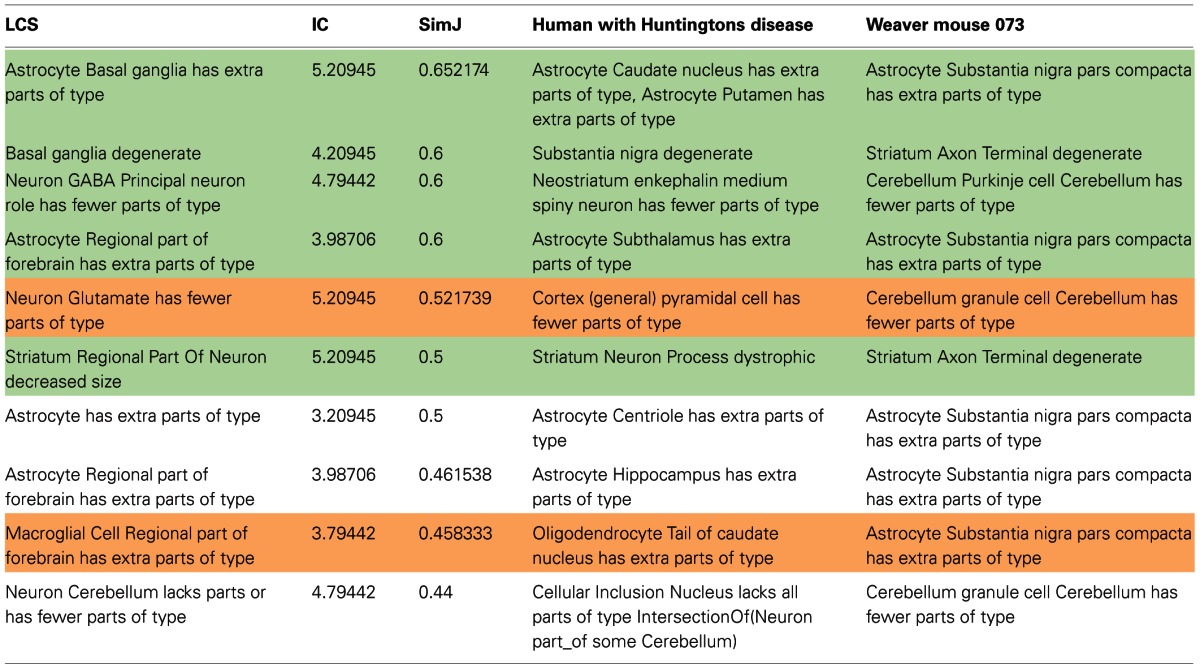
**Comparison of aggregate phenotypes between a representative patient with Huntington's disease and an instance of the weaver mouse (073)**.

*Only the top 10 results, as ranked by similarity score (SimJ) are shown here. Comparisons were evaluated according to whether they were plausible and meaningful (green), plausible but not meaningful (orange), or neither plausible or meaningful (white)*.

The top 10 matches according to overall similarity score are shown in Table 3. Of the 36 matches returned, we ranked 22 to be plausible and useful. By plausible and useful, we mean that someone with relevant expertise would accept the basis of comparison and find it relevant for understanding the commonalities between the two organisms. For example, the most similar match in Table 3 indicates that there is a reactive astrocytosis in different components of the basal ganglia. Although there are different components affected in the two (striatal components in HD; substantia nigra in the weaver mouse), all three nuclei are highly interconnected. Seven out of 36 results were ranked as plausible but not useful. By plausible but not useful, we mean that the basis on which the comparison was returned was sound, but the match was not deemed to provide information that was relevant. For example, the match between a loss of cortical pyramidal neurons (HD) and cerebellar granule cells (weaver) reflects the fact that both are glutamatergic neurons. While true, there are many glutamatergic neurons in the brain, and we currently have no reason to suspect that these particular neuron types share other features or that there is a general vulnerability in glutamatergic neurons in these conditions. In contrast, the match between phenotypes involving degeneration of GABAergic projection neurons was considered both plausible and useful. In this case, a researcher would take note of these phenotypes, in that the two neuron types share both neurotransmitter and projection type and GABAergic projection neurons are not that common in the CNS. Finally, 5 out of 36 matches were deemed neither plausible or useful. Some of these matches involved entities that were not comparable or notable, e.g., that astrocytes had increased numbers of centrioles (HD) and were increased in number in the substantia nigra pars compacta (weaver). It is true that both involved an astrocyte and an increase in number, but the subject and object were not deemed meaningful. In another case, astrocytes were noted to be increased in number in the forebrain, due to an increase in astrocytes in the hippocampus (HD) and in the substantia nigra (weaver). However, as the substantia nigra is not considered a forebrain structure, this reasoning was noted to be in error.

An additional 37 phenotypes for HD were deemed to have no similar phenotypes compared to the weaver mouse (not shown). Of these, 6 were noted as somewhat similar to phenotypes available for the weaver mouse. For example, MEM would have noted the phenotype “Temporal lobe has fewer parts of type dopamine transporter” in the patient with HD as evidence that projections from the mesocortical dopaminergic system may be compromised. This projection arises from dopaminergic cells in the midbrain. However, as of this writing, the knowledge base does not incorporate any knowledge about connectivity among brain regions.

Q4: Which mouse model is the best overall match for a given disease?

The previous two examples provide comparisons of individual phenotypes across organisms. We also investigated whether PhenoSim could reliably measure the strength of overall comparisons between organisms based on the aggregate of their associated phenotypes. The disease view in the PKB interface provides a table with the list of current human diseases modeled in NDPO, and the best predicted match among organisms from PKB for each condition (Table 4), based on a combination of similarity strength and IC. Although PKB contains phenotypes from multiple types of model organisms, the current coverage in PKB is most extensive for mouse models. We thus restricted our analysis to this species.

**Table 4:** **Overall best mouse matches to human disease**.

**Human disease**	**#**	**Best mouse match**	**Mouse description**	**IC**	**SimJ**	**Overall**
Brain ischemia	27	5XFAD transgenic mouse 1	5 A beta mutations	4.18984	0.512734	10.497
Down syndrome	49	PS19 mouse 6 months of age	Mutant tau	4.31912	0.479374	10.5929
Spinal muscular atrophy	49	**Mouse with SMN1 mutated gene**	Mutant survival motor neuron protein 1^*^	4.25612	0.44871	10.4992
Alzheimer's disease	149	**5XFAD transgenic mouse 1**	5 A beta mutations^*^	4.27852	0.417583	10.4905
ALS	70	**SOD1-G93A mouse**	Mutant superoxide dismutase^*^	4.6353	0.389674	10.8194
Huntington's disease	122	**R6/2 mouse 2**	Mutant huntingtin^*^	4.33642	0.371246	10.5021
Pick's disease	72	**PS19 mouse 6 months of age**	Mutant tau	4.35273	0.36528	10.5124
Parkinson's disease	133	**A53T15+ mouse Subject: 252**	Mutant alpha synuclein^*^	4.16459	0.35803	10.317
Progressive supranuclear palsy	50	Weaver mouse 073	Murine mutant	3.73619	0.333625	9.86423
Multiple system atrophy	102	**SOD1-G93A mouse**	Mutant superoxide dismutase	4.28662	0.328572	10.4096
Lewy Body disease	47	A53T15+ mouse Subject: 252	Mutant alpha synuclein	4.03864	0.323762	10.1568

Mouse models in bold are reciprocal matches, i.e., the human with disease is the best match for the mouse model and vice versa. Mouse models that were asserted to be models of the disease by the author of the article from which the phenotypes were taken are indicated by an

* *. #, number of phenotypes recorded in NDPO/PKB*.

An examination of the top mouse match for each disease showed that the results of the phenotype matching were generally successful; that is, the top phenotype matches were judged to be correct, meaningful and useful. For a given disease, PhenoSim ranked matches against animal models that have been developed specifically as models of a particular disease highly (designated by an ∗ in the mouse description column of Table 4). This result is not particularly surprising, as descriptions of the phenotypes associated with the asserted models tend to focus on those pathologies associated with the disease, and thus provide evidence of phenotypes that are similar to those found in human disease.

For those disorders for which PKB did not contain asserted models, PhenoSim still returned organisms that shared a number of significant phenotypic similarities with the human condition. For example, Down Syndrome and Lewy Body Disease were matched against instances of mouse models containing alterations of genes encoding proteins known to be affected in the disorder; MAPT (encoding tau) for Down Syndrome and SNCA (encoding alpha-synuclein) for Lewy Body Disease. Down Syndrome is characterized in later stages by the same types of pathological changes observed in Alzheimer's disease, including the neurofibrillary tangles containing hyperphosphorylated tau protein (Hanger et al., 1991). Lewy Body disease, like Parkinson's disease, is considered a synucleinopathy (Litvan, 1999). The top matches returned for Lewy Body disease include abnormal aggregates of synuclein, a component of Lewy Bodies, in various parts of the neuron and in particular brain regions.

In another case, Progressive Supranuclear Palsy (PSP) was matched against an instance of the Weaver mouse mutant, based on observations that the number of neurons is reduced in the cerebellum and substantia nigra pars compacta (data not shown). Apropos of the example given in the introduction, PhenoSim correctly returned a match between the loss of neuromelanin containing cells in the midbrain of PSP patients and the loss of dopaminergic cells in the midbrain of the weaver mouse mutant. Substantia nigra dopaminergic cells in humans are characterized by the presence of neuromelanin. Note that for both PSP and Down syndrome, the best matches are not reciprocal, that is, while the human with disease matches best to these mouse models, the mouse models have better matches to other conditions.

Not all organism matches were deemed to be successful. MSA is also considered to be a synucleinopathy, but was matched to an instance of the superoxide dismutase (SOD) knockout rather than any of the synuclein mice. Examination of the phenotypes recorded for MSA, however, indicated that the annotators had entered only a few phenotypes that specifically mentioned synuclein, in contrast to Lewy Body Disease. Examination of the top phenotype matches returned from PhenoSim for MSA and the SOD knockout showed matches at a level too generic to be interesting. The top-scoring match in this case was for the presence of increased p25 protein in oligodendrocytes of MSA patients and increased cytochrome oxidase in neurons from the mouse model. The common subsumer leading to the match was *Increase in some protein in nervous system cell*. However, although PhenoSim returned this match as the highest, we note that the SimJ score for the highest match was only 0.52, compared to the top rated matches in the more successful comparisons, which generally were in the 0.8–1.0 range, and the overall similarity was ranked as low. Thus, PhenoSim returned this as the best overall match available from available organisms, not necessarily a good match in an absolute sense. However, we note that the comparison between Lewy Body Disease and the A53T15+ mouse Subject: 252 was also was ranked lower than the other comparisons, but was judged by MEM as having been a successful match. Thus, with the current state of the knowledge base, PhenoSim could not reliably predict the strength of the match based on SimJ or IC scores without examination of the individual phenotype comparisons. We note, however, that when the knowledge base is more fully populated, the reliability of these measures may improve.

## Discussion

Biomedical research is built upon the foundations of model systems that provide both insight into mechanisms of disease and an assay system for testing possible treatments. Matching across these systems at the level of the genes involved is served by several current information systems such as the MGI database. Comparing across systems at the level of phenotype has been more of a challenge because phenotype space is essentially unconstrained (Houle et al., 2010). Phenotypes occur across all scales and temporal dimensions. There is no standard set of tests or assays that reveal them and, until recently, no standard grammar to describe them. Yet comparisons at the level of phenotype likely represent the most critical link for finding relevant systems in which to test therapeutics and for revealing common mechanisms among seemingly unrelated disorders.

Powerful new approaches are starting to be developed to compare phenotypes and gene networks across species, using orthologous phenotypes to identify common genetic networks (McGary et al., 2010). These methods rely on the ability to group similar phenotypes across studies, a process that is confounded in the nervous system because of the multiplicity of nomenclatures for brain regions, classification schemes for neurons, and anatomical scales at which these are studied and compared. The cellular and subcellular signatures of neurological disorders are also quite specific, with subpopulations of cells within a region showing selective vulnerability. Even a general insult like cerebral ischemia disproportionately affects certain cell populations within a subregion (Martone et al., 2000). Thus, a method to identify commonalities among phenotypes within the nervous system along multiple axes would be of significant benefit to unraveling common molecular networks. It is important to note that the ability to compare phenotypes within the nervous system is not just important for identifying potential genetic networks, but also to understand the cellular networks that underlie behavior processes. Thus, even if two organisms do not share a genetic linkage, they may share a behavioral one that reflects the different cellular networks that may contribute to behaviors like motor planning and timing.

We employed formal ontologies as a tool to group and compare phenotypes in neurodegenerative disease, building upon the work of Gkoutos et al. (2005, 2009) and Washington et al. (2009) to define phenotypes using the EQ model. The NIF and other projects have been working with community ontologies to express an information framework for neuroscience, that contains general knowledge about the relationships between brain regions, cell types, cell parts, and macromolecules (Lam et al., 2007; Bug et al., 2008; Hamilton et al., 2012; Imam et al., 2012). Through the definition of a generic template for describing any structural phenotype, regardless of scale, with entities drawn from the NIFSTD ontologies, we showed that we could express structural phenotypes in a way that facilitated cross scale and cross species comparisons between neurodegenerative disease and associated models. We showed that as the underlying core ontologies evolved, the subsequent matches became more nuanced and meaningful.

### Rationale for design

The NDPO/PKB is not designed to function as a diagnostic system, but as a means to facilitate comparing phenotypes, based on knowledge about the entities that were noted in organisms that bore the disease or were developed as models of a disease. We thus modeled structural phenotypes observed in humans that bear a disease, classifying it as a dependent continuant, rather than modeling the disease itself. The assertion of disease as a dependent continuant is more than a mere philosophical exercise. Our concept of diseases as unitary entities is undergoing a significant evolution in the era of personalized medicine, as we focus more on the interactions of pathological processes with disease modifying genes and environmental factors. The assignment of phenotype to organism allows the phenotype to be correlated with any other variable operating at the level of the organism, e.g., genotype, history, and the environment in which the organism lives, without having to carefully model the exact relationships among them. Such relationships are difficult to specify in the case of neurodegenerative diseases, and even more difficult to model explicitly (Gupta et al., 2003; Scheuermann et al., 2009). Although we have not yet implemented the experimental conditions under which the phenotype was observed, the model, as elaborated in Figure 1, easily allows this information to be represented in the knowledge base.

Despite our evolution in views of disease, the concept of disease diagnosis is still highly useful both in a clinical sense and as a means of communicating. In our approach, we balance the need for a canonical vs. more specific, contextual representations not only through the use of the organism, but also through the creation of the NDPO and the PKB respectively. The NDPO is weighted heavily toward exemplar representations of human disease as would be encountered in review articles and represents the most common phenotypes associated with a disease. The PKB, on the other hand, contains phenotype class expressions derived from specific instances of organisms represented in the primary scientific literature or in image data associated with a particular study. The PKB thus provides the mechanism whereby any observed phenotype measured in a human with a disease diagnosis or animal model can be associated with a particular disease, regardless of whether it is considered to be diagnostic for the disease.

Unlike the popular Mammalian Phenotype Ontology, we decompose our phenotype statements so that each entity and quality can be used separately for logical reasoning. The decomposition also provides a flexible language for describing nearly any phenotype at the level of granularity at which it occurs. Such specificity is critical when comparing nervous system phenotypes, because of the multiplicity of cell types within each brain region and the selective targeting of certain neuronal populations. Precomposed ontologies are, by necessity, focused on more general phenotypes. Consider the following annotation of one of the alpha synuclein overexpressing mice in the MGI resource annotated with the MPO: *abnormal myelination, neurodegeneration, axon degeneration, abnormal nervous system morphology, gliosis, astrocytosis, abnormal spinal nerve morphology, alpha-synuclein inclusion body, loss of dopaminergic neurons*. The PKB augments these general features with very specific statements about the type of cell and part of cell affected. It can be envisioned that building the NDPO and PKB using community ontologies like the GO that each phenotype would be able to be classified under the appropriate classes of the MPO.

An advantage of having each entity belong to its own hierarchy is that each phenotype statement can be cross-compared along multiple axes without manually having to assign it to multiple hierarchies. Thus, the statement *tyrosine hydroxylase containing neurons are reduced in the substantia nigra* can be classified based on molecule, cell type, reduction of cell number, and brain region. The richer the relationships within the ontology, the more nuanced the reasoning. By building these statements from community ontologies, the NDPO/PKB benefits from the labor of the community in defining these relationships. For example, the Foundational Model of Anatomy (Rosse and Mejino, 2003), from which NIFSTD draws, recently added the relationships between Brodmann's areas and the cerebral cortex (Turner et al., 2010). Although this makes the system somewhat fluid, it also allows the system to evolve as new knowledge is added without having to reconfigure the system.

PKB and NDPO currently make no attempts to classify changes observed as normal or abnormal, in contrast to the MPO and the HPO. Thus, PhenoSim makes frequent and high scoring matches between phenotypes based on LCS such as *substantia nigra neurons lacks all parts of type cellular inclusions* (normal) and *substantia nigra neurons lacks all parts of type neuromelanin* (abnormal). Matching these two statements clearly provides benefit to a researcher, who would note that the animal model and human disease may not be consistent. However, right now, PhenoSim is using these statements as the basis on which to assert similarity, not distinctions. We are currently considering changes to the model to try to address these types of matches, possibly by including phenotypes associated with organisms that do not bear a disease or utilizing ontologies like the MPO/HPO to provide more explicit declarations of the abnormal state.

As PhenoSim utilizes knowledge encoded in the ontology to improve search and comparison, it is not surprising that PhenoSim performs better than string matching in retrieving relevant results for categories such as GABAergic neuron, cellular inclusion, or pyramidal cell. All matches were returned based on explicit reasoning through common subsumption and the quality of the results clearly depended upon the state of the ontologies. As more specific knowledge was added to the ontology, the common subsumers tended to be more specific and relevant. Many ontologies are built from fairly flat hierarchies followed by the assignment of properties through which additional hierarchies can be generated. Very flat ontologies, e.g., the large list of proteins in the Protein Ontology, generally provide too broad coverage to return meaningful results. In contrast, the current work underway in NIF to provide a knowledge base of neurons based on their properties (Hamilton et al., 2012) allowed PKB to compute common subsumption based on equivalent classes for brain region, transmitter type and projection role.

Although the explicit relationships within the ontology clearly contributed to the quality of the results, an encouraging result was the large number of plausible matches that were returned where a human could clearly draw a relationship between the entities and qualities compared, despite the lack of explicit relationships defined within the ontology. Common subsumers like *change in magnitude relative to some quantity* for *nervous system cell* frequently matched phenotypes involving opposite changes in neuron and glial number. As marked gliosis and activation of microglia are frequently observed in areas that show neuronal or synaptic degeneration, these matches were considered successful, even though the formal reasoning about relationships among glia and neurons is not encoded in the ontology. Because ontologies are labor intensive to construct and by necessity have to be limited in scope, a system that relies exclusively on explicitly encoded or inferred relationships would be of limited utility. However, by specifying some basic top down principles, e.g., the relationships among different qualities related to *decreased*, major classes of biological entities and a brain partonomy that could be used to group related structures, the system performed better than would be likely on string matching alone. These results also support the findings of Dahdul et al. (2010) that even a course level of annotation using the PATO qualifiers can improve the ability of an information system to group phenotypes.

Because the base ontologies drew relationships between closely related entities and qualities, the use of ontologies for constructing phenotype descriptions was also able to mitigate somewhat against the variety of ways in which authors described similar phenotypes and inconsistency of different annotators in choosing appropriate qualities and terms to apply to data. Such inconsistency is common in any system, and reflects different styles and expertise among annotators, the flexibility of language, human error and the inevitable evolution of annotation standards during the course of developing and populating an information system (Dahdul et al., 2010; Turinsky et al., 2010).

The conditions under which the knowledge base was constructed, including using students who were only modestly trained in curation and ontology construction, was meant to mimic a situation in which individuals of different levels of expertise would be contributing to the knowledge base. In the current state, a contributor has to have some training in OWL and ontology editors. However, because the phenotype statements rely on a common template, we can envision the creation of image and literature annotation tools that allow researchers to construct structured phenotype statements without formal training. The aggregation of phenotypes from different laboratories working on common strains would be facilitated through proper identification of the organisms used in a study through the use of unique identifiers supplied by the model organism databases.

### Temporal progression

Neurodegenerative diseases are characterized by a long progression, typically manifesting themselves symptomatically in humans late in maturity and well after the disease process is underway. Phenotypes associated with the disease process may change depending upon the stage of the disease and the age of the organism. For researchers trying to select a model system or to understand underlying disease processes, the age at which a phenotype is present is a critical piece of information. As a partial solution, we constructed temporal slices of organisms, essentially noting phenotypes associated with an organism of a particular age or age range. These temporal slices clearly indicate that a phenotype occurs at a given time point, but because they are not connected to each other through temporal relationships, we cannot perform any reasoning. Neither do we capture temporal characteristics likely to be important in comparing models and disease, e.g., whether the pathological insult is chronic or acute. Future plans include the use of Allen interval calculus (1983) to capture these temporal relationships so that temporal relationships among phenotypes can be inferred and used as a basis of comparison among organisms.

## Conclusions

We have implemented the EQ model to address the challenging problem of phenotype representation of neurodegenerative disease and associated models. The initial results suggest that the knowledge-based approach for expressing and comparing multi-scale phenotypes is promising and that providing a tool like PKB for computing and browsing similar phenotypes may have utility for identifying relevant animal models and commonalities among phenotypes. The current knowledge base was not designed for nor sufficiently populated to be used for unbiased quantitative evaluation of phenotypic similarity. Indeed, the tools and approach were constructed with the goal of providing researchers working independently a standard format for reporting on phenotypes within published studies and imaging to aid in search and comparison. A reasonable question would be whether population of a common database through independent contributions would ever lead to a resource that would be of sufficient breadth and depth for deeper data mining, or whether a more systematic strategy for population is required, as is the case with most “omics” approaches (Cachat et al., 2012). However, because phenotype space is unconstrained, we feel that exploration of more efficient and computable methods for representing the types of phenotypes published every day in the scientific literature would aid in more effective utilization of the information we have available and allow more effective identification of commonalities and differences. Future work will be directed toward refining the comparison metrics to determine whether reliable ranking methods can be developed that would facilitate identification of common genetic pathways.

**Figure 4:**
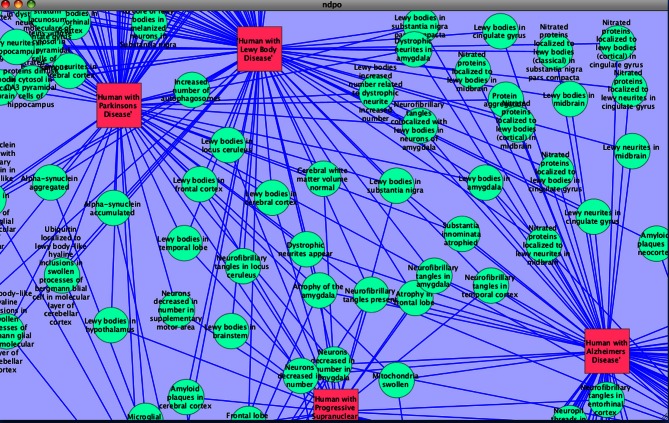
**Graphical representation of relationships among human disease phenotypes created using Cytoscape network visualization tool (http://cytoscape.org/)**.

As the amount of neuroscience data and discoveries related to disease multiplies, it becomes increasingly important to express this data and knowledge within a common framework (Akil et al., 2011). The NIF was developed specifically for that purpose for neuroscience via the NIFSTD ontologies. The NIFSTD recognizes that to connect the various subdisciplines and scales of study for the nervous system requires some level of formal semantics as a way of organizing and comparing data. The goal of NIF is thus to provide researchers with the building blocks in which to create new data and knowledge in a way that promotes interoperability, not to restrict expression (Bug et al., 2008; Imam et al., 2012). The ontologies that form the building blocks of the NDPO/PKB essentially provide us with a simplified yet powerful language for composing phenotype statements in a way that exposes them to the power of algorithmic tools for comparison, similar to the array of tools for pathway analysis among molecular entities or sequence comparison tools among thousands of sequences genotypes (Figure 4). As the underlying ontologies continue to evolve, we expect that algorithms will perform better, aiding basic and clinical researchers interested in selecting appropriate models and in exploiting the knowledge base to derive new hypotheses about the molecular underpinnings of disease.

### Conflict of interest statement

The authors declare that the research was conducted in the absence of any commercial or financial relationships that could be construed as a potential conflict of interest.

## Footnotes

^1^For references to terms that appear in an ontology, we supply the ontology name, e.g., Gene Ontology and the unique identifier for the class name.
